# A Generalizable Brain-Computer Interface (BCI) Using Machine Learning for Feature Discovery

**DOI:** 10.1371/journal.pone.0131328

**Published:** 2015-06-26

**Authors:** Ewan S. Nurse, Philippa J. Karoly, David B. Grayden, Dean R. Freestone

**Affiliations:** 1 NeuroEngineering Laboratory, Department of Electrical & Electronic Engineering, The University of Melbourne, Melbourne, VIC, Australia, 3010; 2 Centre for Neural Engineering, The University of Melbourne, Melbourne, VIC, Australia, 3010; 3 Department of Medicine St. Vincent’s Hospital Melbourne, The University of Melbourne, Fitzroy, VIC, Australia, 3065; 4 Department of Statistics, Columbia University, New York, New York, USA, 10027; Duke University, UNITED STATES

## Abstract

This work describes a generalized method for classifying motor-related neural signals for a brain-computer interface (BCI), based on a stochastic machine learning method. The method differs from the various feature extraction and selection techniques employed in many other BCI systems. The classifier does not use extensive *a-priori* information, resulting in reduced reliance on highly specific domain knowledge. Instead of pre-defining features, the time-domain signal is input to a population of multi-layer perceptrons (MLPs) in order to perform a stochastic search for the best structure. The results showed that the average performance of the new algorithm outperformed other published methods using the Berlin BCI IV (2008) competition dataset and was comparable to the best results in the Berlin BCI II (2002–3) competition dataset. The new method was also applied to electroencephalography (EEG) data recorded from five subjects undertaking a hand squeeze task and demonstrated high levels of accuracy with a mean classification accuracy of 78.9% after five-fold cross-validation. Our new approach has been shown to give accurate results across different motor tasks and signal types as well as between subjects.

## Introduction

A brain-computer interface (BCI) is a system that measures central nervous system activity and converts it into an artificial output, such as movement of a cursor or robotic limb, thereby providing an alternative pathway for the brain to interact with its environment [1]. Typically, the signal that is used for BCIs is a measurement of the electrical or magnetic fields of the brain [2]. A grand challenge in BCI research and development, along with more general fields in neuroscience, is developing methods to decode a user’s intent from the neural signals. An ideal solution would be a method using a portable, non-invasive recording system, such as electroencephalography (EEG). This is an extremely difficult problem given that the EEG signal recorded at the scalp is an abstraction of the underlying neural activity [3].

The scalp EEG is a measure of mean electrical activity from large populations of cortical neurons. Thus, the causal relationship between EEG activity and movement is not well understood [4]. In order to decode a user’s intended movement, signal features correlated to activity are extracted; however, finding optimal, task-specific features is an ongoing focus of BCI research [2, 5]. Given the complexity of the underlying neural data and the large number of potentially useful features, a more generalized approach to feature extraction will be valuable. The two key methodologies applied to transform neural data for BCIs are classification and regression. The work in the current study is focused on the classification problem, whereby features are used to distinguish between discrete classes of control signal. The optimal feature choice is highly dependent on the specific task [6]. For instance, features used for motor imagery-based tasks, such as alpha and beta event-related desynchronization may provide variable accuracy for different movement tasks [7]. Furthermore, it is unclear if particular features will generalize well across different users performing the same task. For example, it has been shown that motor imagery-specific features provide variable accuracy for BCI control, with some users unable to reliably produce distinguishable oscillations [8, 9]. Therefore, the development of a subject-specific BCI that does not rely on an *a-priori* choice of features will represent a significant advance in the field.

Challenges in finding optimal features have inspired alternative approaches to feature extraction and classification without extensive hand-coding in an attempt to move away from reliance on domain-specific knowledge. Various optimization methods and search heuristics have been utilized to develop suitable sets of features. For instance, search heuristics have been employed to produce a suitable subset from candidate sets of potential known features [10, 11]. There is recent evidence that it is viable to classify neural signals without requiring *a-priori* knowledge and instead allow machine learning techniques to infer useful features [12, 13]. This approach is appealing for increasing the generalizability of a classifier between subjects and across multiple signal types or applications. Given the complexity of neural signal generation and the inability to pre-define and describe all potentially useful features for a given task, a new approach to classification is well worth pursuing. This study addresses this challenge by developing a method that can be used in subject-specific BCIs, without constraining the domain of the feature space.

The general concept of the algorithm presented in this paper was inspired by how evolution has driven the development of the human brain to extract features from the environment, and to learn patterns of these features to discriminate information. With this idea in mind, we see this algorithm as a method for creating an extension of the subject’s brain that is dedicated to decoding the user’s neural activity for a particular application. In order to achieve this goal, an artificial neural network (ANN) was used to extract subject-specific features of the data to classify the user’s actions. An ANN is a machine learning tool that loosely mimics one way the brain adapts and classifies input patterns based on reinforcement learning [14]. A genetic algorithm (GA) was used as a representative of a stochastic search algorithm to constrain the ANNs. The GA was applied to select the number of hidden layers and number of neurons per layer of the ANN for given training data.

This article describes a novel method for classifying neural signals (such as EEG signals) with applications in BCIs. The novelty of the method is that it requires no pre-determined features in order to perform classification. Instead, the neural time-series is passed directly into an ANN that has a structure that is constrained by a random search (in this case a GA). In this way, a general purpose BCI classification algorithm is created that alleviates reliance on *a-priori* assumptions about the data.

This paper is structured as follows. First, we describe the algorithm architecture in terms of its components, which are a GA and a population of ANNs. Next, we describe the data that was used for testing the performance of the new method, which consist of publicly available datasets and new experimental data collected specifically for this study. Then, the results are presented as well as a group analysis demonstrating the viability of this method. Finally the significance of the study is discussed in the context of BCIs, along with recommendations for future developments.

## Methods

**Fig 1:**
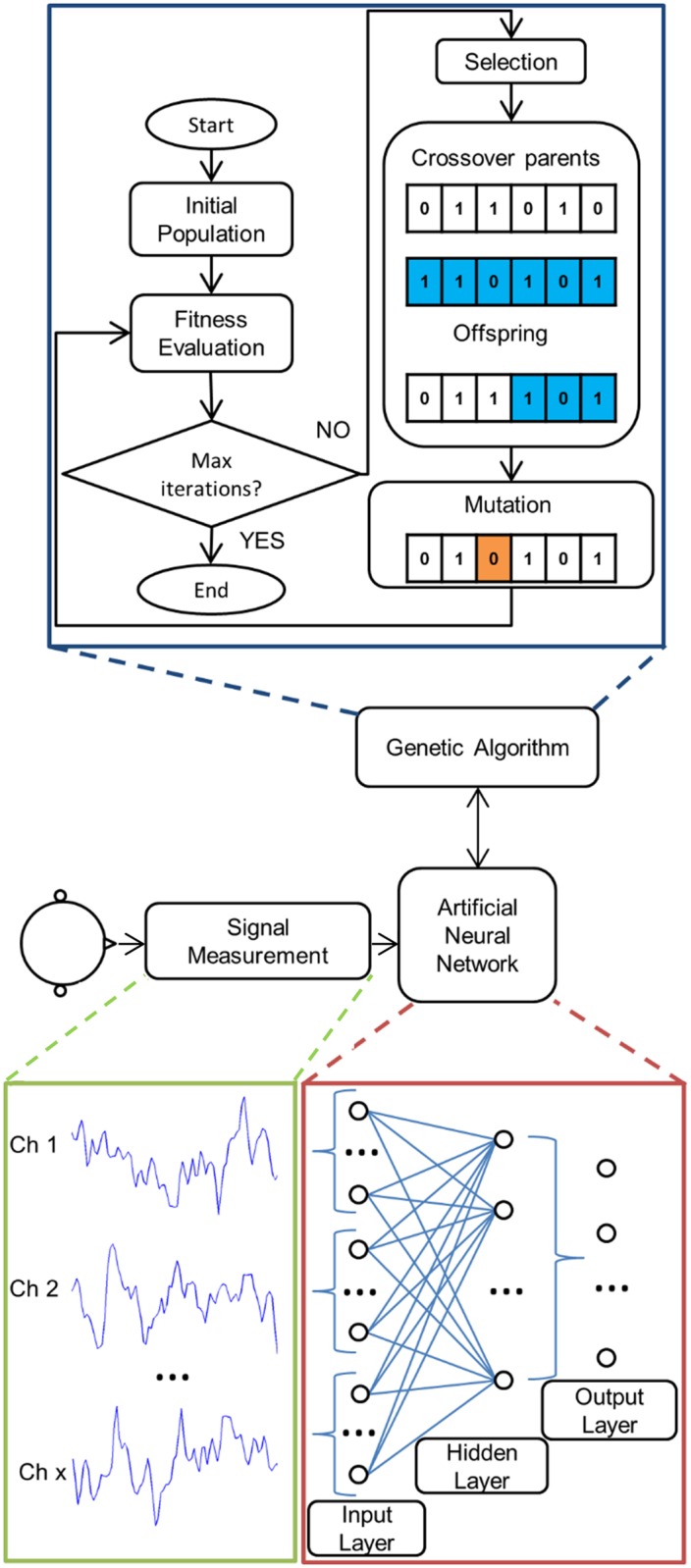
Illustration of method. Signals acquired from the brain-computer interface (BCI) user are initially used to train an artificial neural network (ANN). The number of hidden layers and neurons is determined using a genetic algorithm (GA). At the termination of the GA, the network found to have the fittest structure is used in the BCI. The ANN is a fully-interconnected multi-layer perceptron. The input layer consists of every time-point of each channel. Hence, each neuron in the first hidden layer is able to generate features based on both spatial and temporal inferences. The hidden layers then feed into the output neurons, which determine the classifier output.

This section describes the three key components of our method; artificial neural networks (ANNs), the genetic algorithm (GA), and neural data acquisition. Fig 1 is a schematic overview of the algorithm used to identify features and perform classification of neural data. Table 1 lists the parameters used to initialize and run the algorithm. Fig 1 shows that within the GA, a population of ANNs performed feature extraction and classification on a window of time-series neural data. That is, the input to the first layer is the raw time-series EEG. The features that are extracted by the ANNs are dictated by the weights of the neurons in each network, which are updated via backpropagation. The weights for every time point of the input can be thought of as a filter that is tuned during network training to find frequency bands and electrodes containing task-related information. The possible feature space is governed by the number of samples in the window, the connectivity structure of the network, the number of layers, and the number of neurons in each layer.

**Table 1:** Algorithm parameters for artificial neural network (ANN) and genetic algorithm (GA).

Parameter	Competition Data	Hand-Squeeze Data
GA Population Size	30	30
No. of Generations	20	20
No. of Elites	1	1
Mutation Rate	2%	2%
SA Iterations	10	10
ANN maximum layers	3	3
ANN maximum neurons per layer	500	500
ANN epoch length	1 s (BCI IV) 0.5 s (BCI II)	0.4 s
Data sample frequency	400 Hz (BCI IV) 100 Hz (BCI II)	250 Hz

The parameters used to initialize and run the algorithm for the competition data sets and hand-squeeze task. (Note that SA refers to the simulated annealing augmented backpropagation used for ANN training.)

### Artificial Neural Network

Fully interconnected multi-layer perceptrons (MLPs) with a hyperbolic tangent activation function constituted the candidate ANNs [15]. The input was the time-domain neural signal and the output was the classification of the user’s action. The mean of each epoch was subtracted before it was used as network input. The signal from each channel was not standardized to unit variance, as it was anticipated that the variance would contain considerable task-related information. The impedance of all channels was maintained below 10 kΩ to ensure variance due to noise was minimized. The number of neurons in each hidden layer and the number of hidden layers were selected by the GA. MLPs had between one and three hidden layers, with a maximum of 500 neurons in each hidden layer (see Table 1). The input layer was configured so that, for each electrode, every time point of each epoch fed into every neuron, as shown in Fig 1. This enables features to be extracted in both space and time. Output class was determined by the maximum of the output neurons. Each network was initially trained using scaled conjugate gradient backpropagation [16], which was implemented in MATLAB (Mathworks, version 2012b) using the ‘patternnet’ and ‘trainscg’ functions for network initialization and training, respectively. The scaled conjugate gradient backpropagation for network training was augmented using simulated annealing.

Simulated annealing was used to reduce the probability of converging to a local minima during network training, by slowing down the learning of the standard backpropagation algorithm. After backpropagation training, each weight, *w*, was adjusted by adding a randomly selected value from the uniform distribution [−*w*, *w*]. The network was then re-trained by backpropagation using the new weights. The re-trained network was adopted if the classification performance on the unseen validation set improved. If performance decreased, the network was adopted with exponentially decreasing probability for each iteration of the simulated annealing. This process was repeated for 10 iterations, which gave a balance between computational burden and improved performance.

Network performance was measured using the Cohen’s kappa value [17] (see S1 Text for definition). Cohen’s kappa takes into account not only the accuracy of the classifier but also the proportion of the accuracy that is due to chance [18]. The same metric was used as a fitness function for the GA.

### Genetic Algorithm

The population of the GA was made up of candidate ANN classifiers. The genome of each classifier was represented by a binary string that defined the number of neurons in each hidden layer and the number of hidden layers for the network. Reproduction of the individual classifiers in the population was through a process of roulette wheel style crossover [19]. The new individual was formed by a single-point crossover of the genomes of the two individuals, as shown in Fig 1. There was a 2% chance of a mutation occurring in each ANN, which was implemented by flipping one randomly selected bit in the binary genome. The fitness of the new population was then calculated as the Cohen’s kappa value of the unseen validation dataset used after network training, and the cycle continued. In this way, the GA constrained the development of the ANNs to a higher performing region of the solution space.

The population of candidate networks was initialized such that there were approximately equal numbers of one, two and three hidden layer networks in the initial population. The GA was run for 20 generations with a population of 30 candidate networks. In each iteration, only the fittest network (the elite) was retained for the next generation. At the termination of the GA, a final neural network with an optimized number of neurons and hidden layers was used to extract features from neural data and classify previously unseen data.

### Competition Data

Two existing open access brain-computer interface (BCI) competition datasets were used to assess the performance of the new algorithm. The competition data was from BCI IV data set three [20] and BCI II dataset four [21]. BCI IV data set three consists of a ten channel magnetoencephalogram (MEG) recording (sampled at 400 Hz) taken from two participants as they performed wrist movements in one of four directions using a joystick. The data set contains 40 trials for each of the four classes of movement (up, down, left or right), each one second in duration. The number of trials in the test set were unequal but similar. BCI II data set four consists of a 28 channel EEG recording from one subject (sampled at 100 Hz) undertaking a keyboard button-press task with the index finger of each hand. The task was to determine which hand was being used. The data set contains 316 trials for each of the two classes of movement (left or right hand), each 500 ms in duration. Both BCI competition datasets were divided into predefined training and testing sets and the same sets were used to test the current algorithm in order to compare the performance with previous results.

### Data Acquisition

In addition to validation on the competition datasets, a new dataset collected specifically for this research was used to further evaluate algorithm performance.

#### Ethics Statement

New EEG data was collected with ethics approval (ID 1339680, Human Research Ethics Committee, University of Melbourne) from five participants (A-E) who performed a self-paced hand squeeze task. Written consent was obtained from all participants. The participants were right handed, with the exception of Participant E. None of the participants had previous background experience with BCIs.

EEG data were acquired using a SynAmps2 64 channel Quik-Cap and a SynAmps2 24 bit amplification system (Compumedics Ltd), although not all electrodes were used for all participants. The times of movement were time-locked to the EEG by simultaneously collecting electromyography (EMG) using the same acquisition system. The surface EMG electrodes were attached to the flexor digitorum on the forearms. Data was sampled at 1 kHz, bandpass filtered between 0.1–100 Hz and notch filtered at 50 Hz using a windowing method. EEG and EMG data was first tapered using a Hamming window then filtered with a zero-phase delay FIR filter (Hann window), which was implemented in CURRY 7 (Compumedics Ltd). Participants were seated and asked to alternate clenching their left and right hands at their own pace with their eyes open, focusing their eyes on a point on the wall. No stimulus was given to prompt the participant to clench their hand in order to minimize the influence of visual or auditory evoked potentials in the training data. In each hand, the participants held a foam squeeze-ball. Recordings were undertaken in four minute sessions, with rests between each session. Typically, data was recorded for a total of 20–30 minutes. Recorded EEG data were decimated to 250 Hz prior to training the network. Data collected is available at https://github.com/EwanNurse/A-Generalizable-BCI-using-Machine-Learning-for-Feature-Discovery.

Movement detection was achieved by extracting the RMS power from the EMG signal in order to label the EEG data into the various classes. RMS power was calculated in sliding windows of 500 ms. When the RMS value exceeded a manually set threshold, a movement was detected. This subject-specific threshold was determined prior to the trial start by visual inspection of the baseline RMS power compared to during hand-clenches. Participants were instructed to maintain similar hand-squeeze motions and keep their arm still between each action to ensure the threshold was consistent. Using the EMG labels, the EEG data was separated into epochs, 100 ms before the onset of clenching and ending 300 ms after the onset of clenching. A refractory period of 300 ms was applied after a clench was detected to prevent a single clench being detected twice. Time periods in between hand squeezes were also used as examples of no-movement signals; i.e., times when the participant was not clenching either hand for at least 500 ms. The same window length (400 ms) was used for the no-movement EEG epochs. Two types of classifier were created with this data set: a two-class classifier that detected either a left or right hand movement and a three-class classifier that also included a no-movement state.

A method of five-fold cross validation was implemented to evaluate the performance of the two-class and three-class methods of classifier. Each iteration of the five-fold cross validation consisted of a different randomly allocated unseen test set (20% of the data) and training set (80% of the data). The test sets were not used during the validation of ANN training. For each test set the classification performance was measured in terms of the Cohen’s kappa value and accuracy. A *p*-value for each participant was then calculated from their kappa scores to determine the statistical significance of the results [17]. The number of training examples obtained for left hand squeezes, right hand squeezes, and no-movement for all subjects are shown in Table 2. It can be seen that the number of left and right epochs is well balanced, and hence is more likely to produce an unbiased ANN. More no-movement epochs were generated as it was supposed that more examples would be required to produce a representative set.

**Table 2:** Recorded data epochs.

Subject	Left	Right	No-movement
A	368	398	602
B	317	329	731
C	326	321	657
D	546	563	759
E	507	499	926

The total number of data epochs recorded for each subject that were used as artificial neural network training and test inputs for left and right hand-squeeze and no-movement signal classes

## Results

### Simulated Annealing

**Fig 2:**
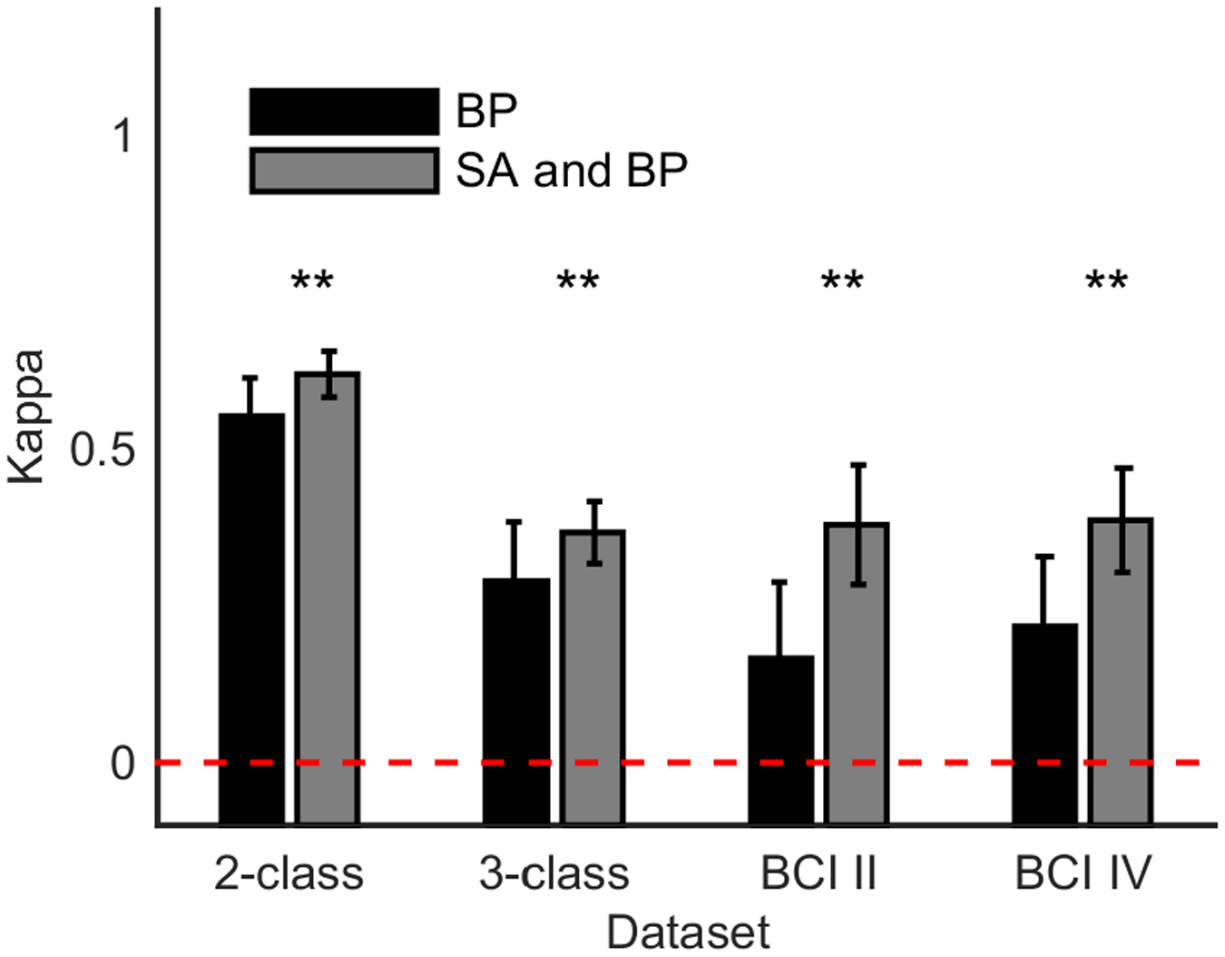
Classification performance of an artificial neural network (ANN) using backpropagation (BP) and simulated annealing augmented backpropagation (SA). Two randomly generated one-, two- or three-layer ANNs were created. Both ANNs had the same number of hidden layers and neurons. Each ANN was then trained using either BP or SA and tested on the same unseen test set. This process was repeated 50 times. The bar represents the mean Cohen’s kappa score for each group and the error bars are the standard deviation of the 50 kappa scores. The outcome for chance performance is shown as a red dashed line (kappa score of zero). The asterisks indicate the confidence level with which to reject the hypothesis that the two bars are from the same distribution using a two-tailed *t*-test (** for *p* < 0.01). For exact *p*-values see S2 Appendex. Datasets are from the BCI II competition, dataset 4 and the BCI IV competition, dataset 3. The results for the two- and three-class hand-squeeze datasets are taken as the average values across five participants.

In order to verify the benefits of using SA in the classification algorithm, the performance of a network trained using scaled conjugate backpropagation with and without SA was assessed. A randomly generated one-, two- or three-layer network was trained on the data using both backpropagation and SA augmented backpropagation, and then its Cohen’s kappa score was evaluated on an unseen test set. This process was repeated for 50 realizations, using 10 iterations of SA. A two-tail *t*-test was used to determine whether the two methods could be considered as significantly different. Fig 2 shows the results of this comparison for both competition datasets and the two- and three-class hand squeeze task. It can be seen from Fig 2 that the standard deviation of the Cohen’s kappa score decreased with the use of SA-modified backpropagation and the mean performance significantly increased, for every dataset. However, the improvement appears more pronounced for datasets that contained less data (the BCI competitions).

### Competition Datasets

**Fig 3:**
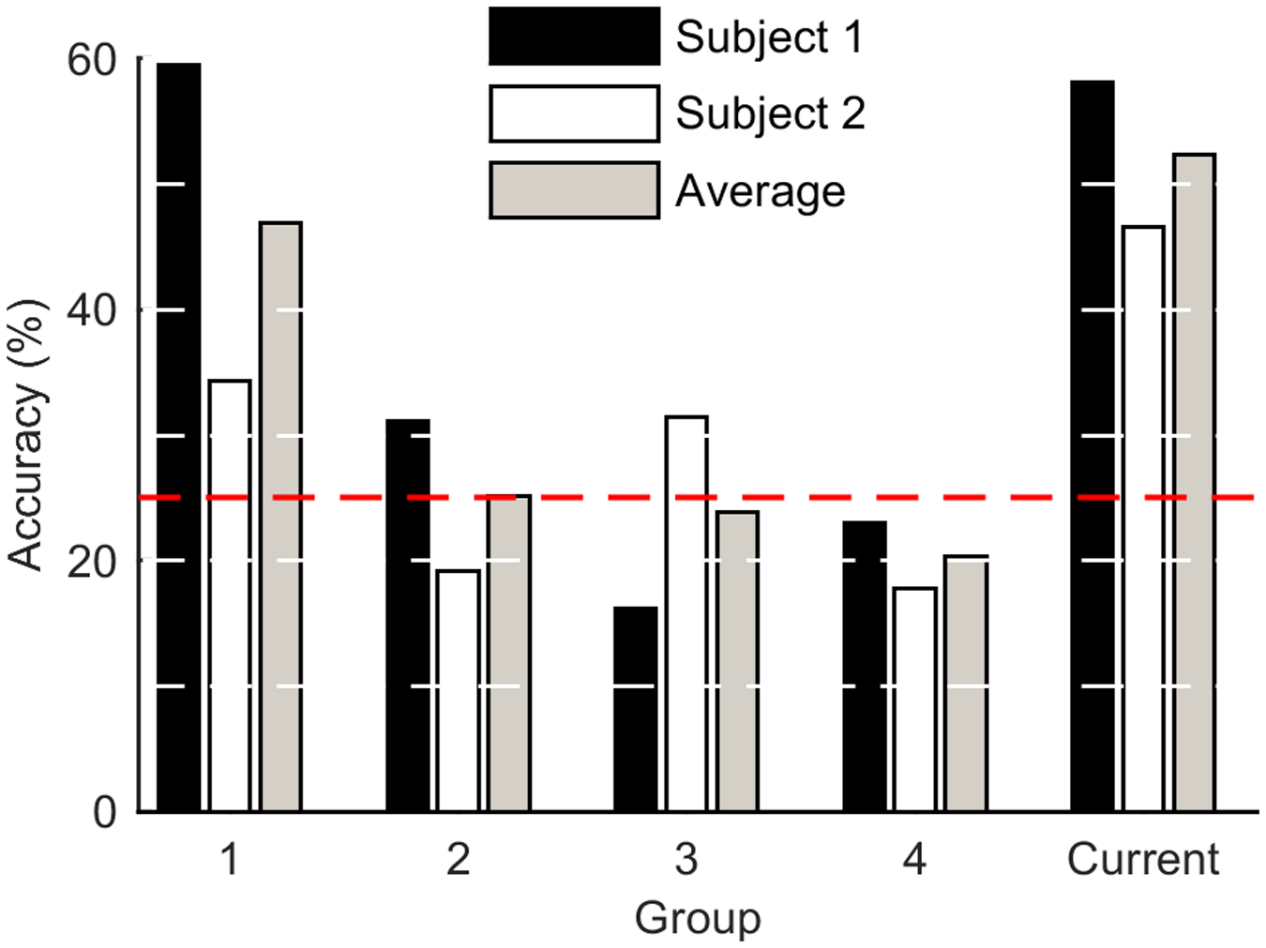
Results for BCI IV data set 3. The classification accuracy for the current study is compared to previous work by (1) Hajipour et al. (2) Li et al. (3) Montazeri et al. (4) Want et al. The red dashed line represents chance outcome (25%). White dashed lines indicate minor gridlines.

**Fig 4:**
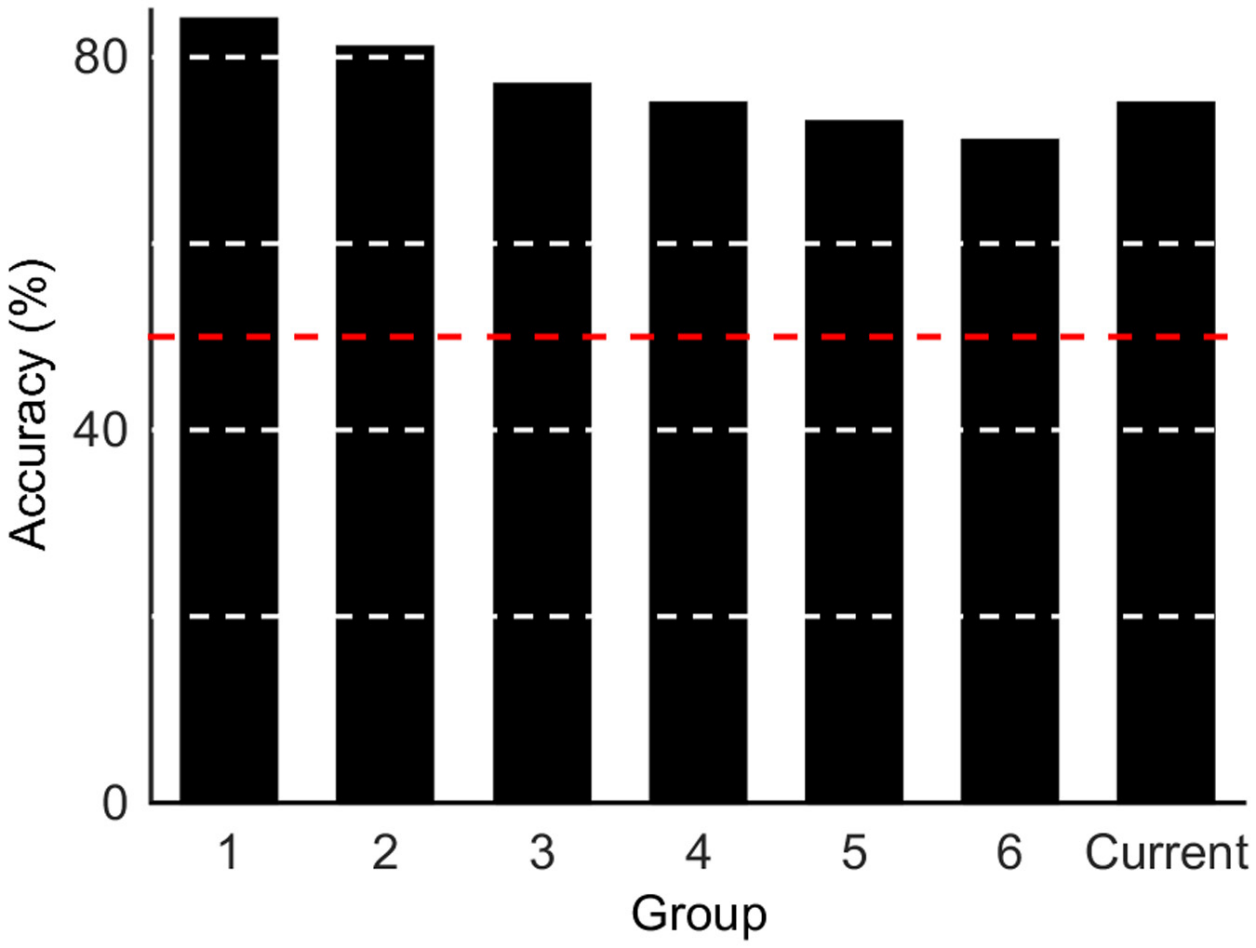
Results for BCI II data set 4. The classification accuracy for the current study is compared to previous work by (1) Zhang et al. (2) Neal (3) Hoffmann (4) Huang et al. (5) Mensh (6) Brugger et al. Only the top 6 competition entrants are shown. The red dashed line represents chance outcome (50%). White dashed lines indicate minor gridlines.

The output accuracy for the BCI IV dataset three is shown in Fig 3. These results are reported in terms of accuracy as the information needed to determine Cohen’s kappa was not available. Fig 3 shows the results for each subject and the averaged result from the best network for our algorithm alongside the competition results [20]. The classification accuracy for the current study was 58.1% and 46.6% for Subject 1 and 2, respectively (average of 52.4%). This is higher than three of the previous published results for both datasets, and approximately equal to the winning entrant (59.5% and 34.3% for subjects 1 and 2, with an average of 46.9%). The same process was repeated for the BCI II data set four. The classification results are shown in Fig 4. The performance of our system was comparable to other competition entrants, with 75% accuracy.

### Hand-Squeeze Task

**Fig 5:**
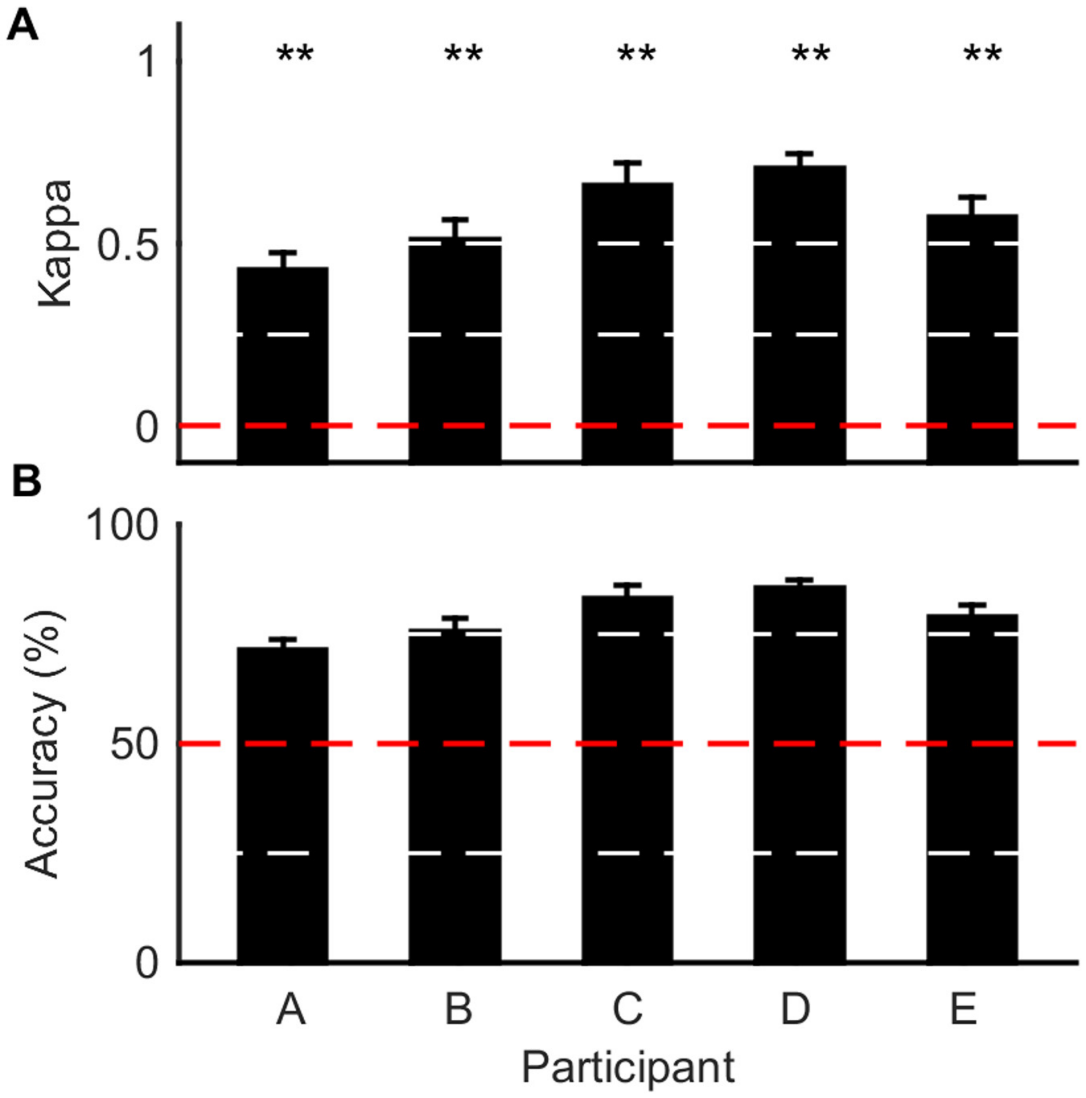
Performance of two-class hand squeeze classifier. The classification performance for the current method is shown as the average performance across the five-fold cross validation for each participant. **A** The average Cohen’s kappa score on unseen test sets. Error bars are standard deviation across 5 folds. The red dashed line shows chance performance (kappa = 0). White dashed lines indicate minor gridlines. The asterisks represent the confidence level to reject the null hypothesis that the results are not significantly different to chance performance (** for *p* < 0.01) using the kappa significance test (described in S1 Text). For the exact *p*-values see S2 Appendix. **B** The average percentage classification accuracy on the unseen test sets. The error bars are standard deviation after five-fold cross validation. The red dashed line represents chance outcome (50%). White dashed lines indicate minor gridlines.

**Fig 6:**
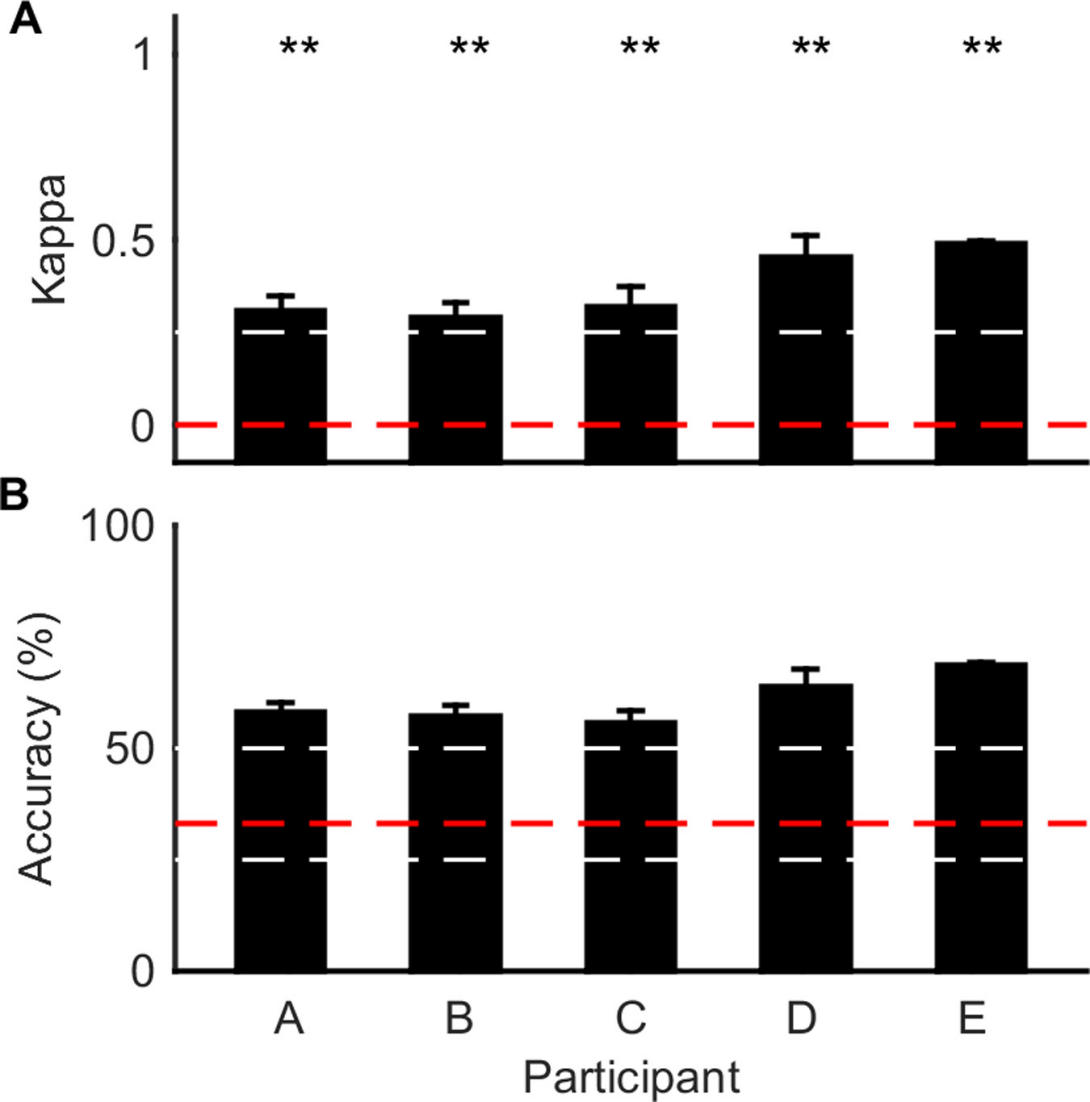
Performance of three-class hand squeeze classifier. The classification performance for the current method is shown as the average performance across the five-fold cross validation for each participant. **A** The average Cohen’s kappa score on unseen test sets. Error bars are standard deviation after five folds of cross validation. The red dashed line shows chance performance (kappa = 0). The asterisks represent the confidence level to reject the null hypothesis that the results are not significantly different to chance performance (** for *p* < 0.01) using the kappa significance test (described in S1 Text). For the exact *p*-values see S2 Appendix. **B** The average percentage classification accuracy on the unseen test sets. The error bars are standard deviation after five-fold cross validation. The red dashed line represents chance outcome (33%). White dashed lines indicate minor gridlines.

A plot of the cross-validated classification results for the two-class and three-class methods are shown in Figs 5 and 6, respectively. Across the five participants, the two-class classifier gave an average kappa of 0.58 and an average accuracy of 78.9%. The three-class classifier gave an average kappa of 0.37 and an accuracy of 60.7%. Both of these are well above the chance performance, which would give kappa values of zero for both methods. Figs 5 and 6 show that all the results were significantly above chance performance for all participants (*p* < 0.01) using the kappa significance test (described in S1 Text).

**Fig 7:**
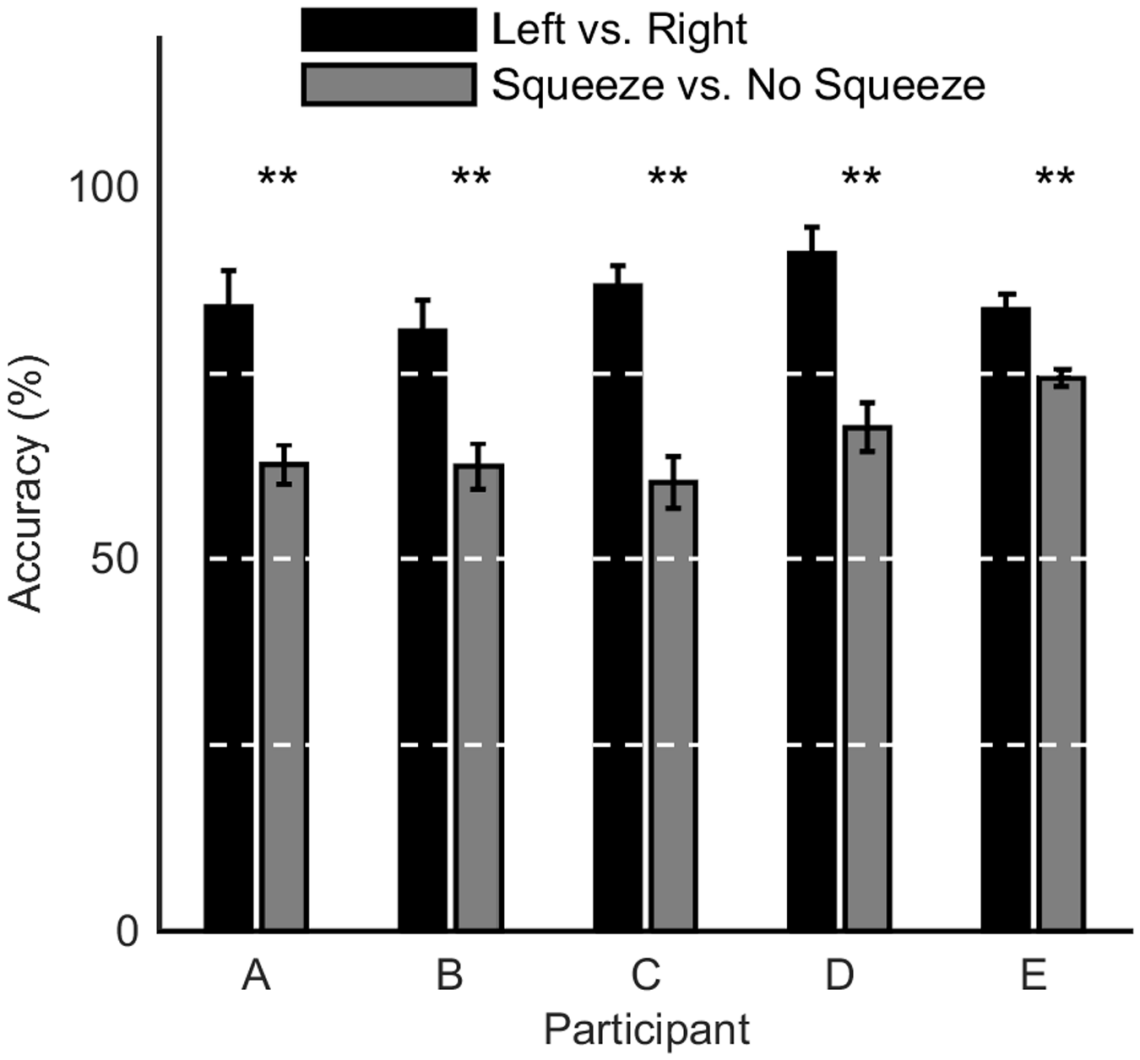
Comparison of accuracy between hand-squeeze detection and left vs. right detection of three-class hand squeeze classifier. The first bar for each participant is the accuracy for correct classification of a hand squeeze (left or right), given that a hand squeeze had occurred. The second bar for each participant is the accuracy for detection of a hand squeeze regardless of laterality. The bars show the mean accuracy across the resulting confusion matrices after five-fold cross validation for each participant. The error bars are the standard deviation over the five confusion matrices. The asterisks represent the confidence level to reject the null hypothesis that the classification accuracy once a hand squeeze is detected is significantly different from the accuracy of detecting if a hand squeeze has occurred using a two-tailed *t*-test (** for *p* < 0.01). White dashed lines indicate minor gridlines. For the exact *p*-values see S2 Appendix. For the full confusion matrices, see S1 Appendix.

Fig 7 shows that, for all participants, the three-class classifier was significantly better at distinguishing between left and right hand squeezes (once a hand squeeze had been correctly identified) than it was at detecting whether a hand-squeeze had occurred or not. A two-tailed *t*-test was used to determine the confidence level to reject the null hypothesis that the classification accuracies were not significantly different (using the five-fold cross validation results). It should be noted that although both the two- and three-class classifiers used the same datasets, there are well known difficulties in comparing the results of classifiers with different numbers of classes, such as uneven probability distributions for different classes [18, 22].

### Neural Network Analysis

**Fig 8:**
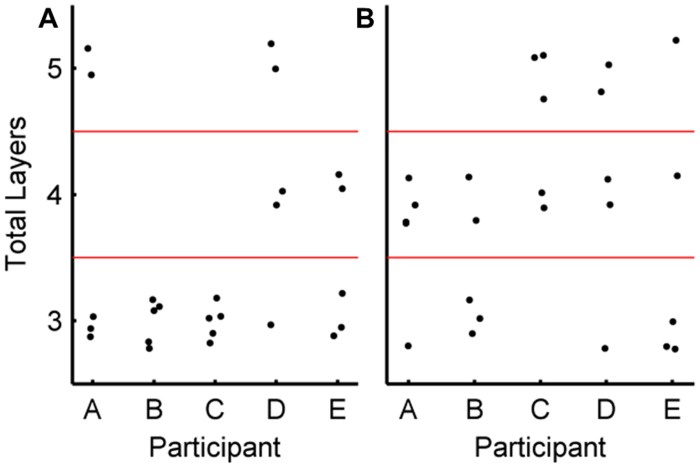
Number of layers of the artificial neural networks (ANNs). The total number of layers in the final ANN classifier after each fold of cross-validation for every participant. A dot is placed in the relevant row for each classifier, where the number of hidden layers is two less than the total number of layers, since there is always an input and output layer. **A** Results for two-class dataset. **B** Results for three-class dataset. The networks trained on the three-class dataset have a higher median number of layers than the networks trained on two-class data (Wilcoxon rank sum test, *p* = 0.0094).

Fig 8 shows the number of layers in the final ANN for each participant over the five-fold cross validation. It can be seen that there was no clear pattern to the number of hidden layers that were selected; however, there was a tendency for more hidden layers to be used in the three-class problem compared with the two-class problem (Wilcoxon rank sum test, *p* = 0.0094). The number of neurons for each hidden layer are presented in S1 Appendix. The results do not demonstrate a consistent pattern in the number of neurons chosen for a given participant.

**Fig 9:**
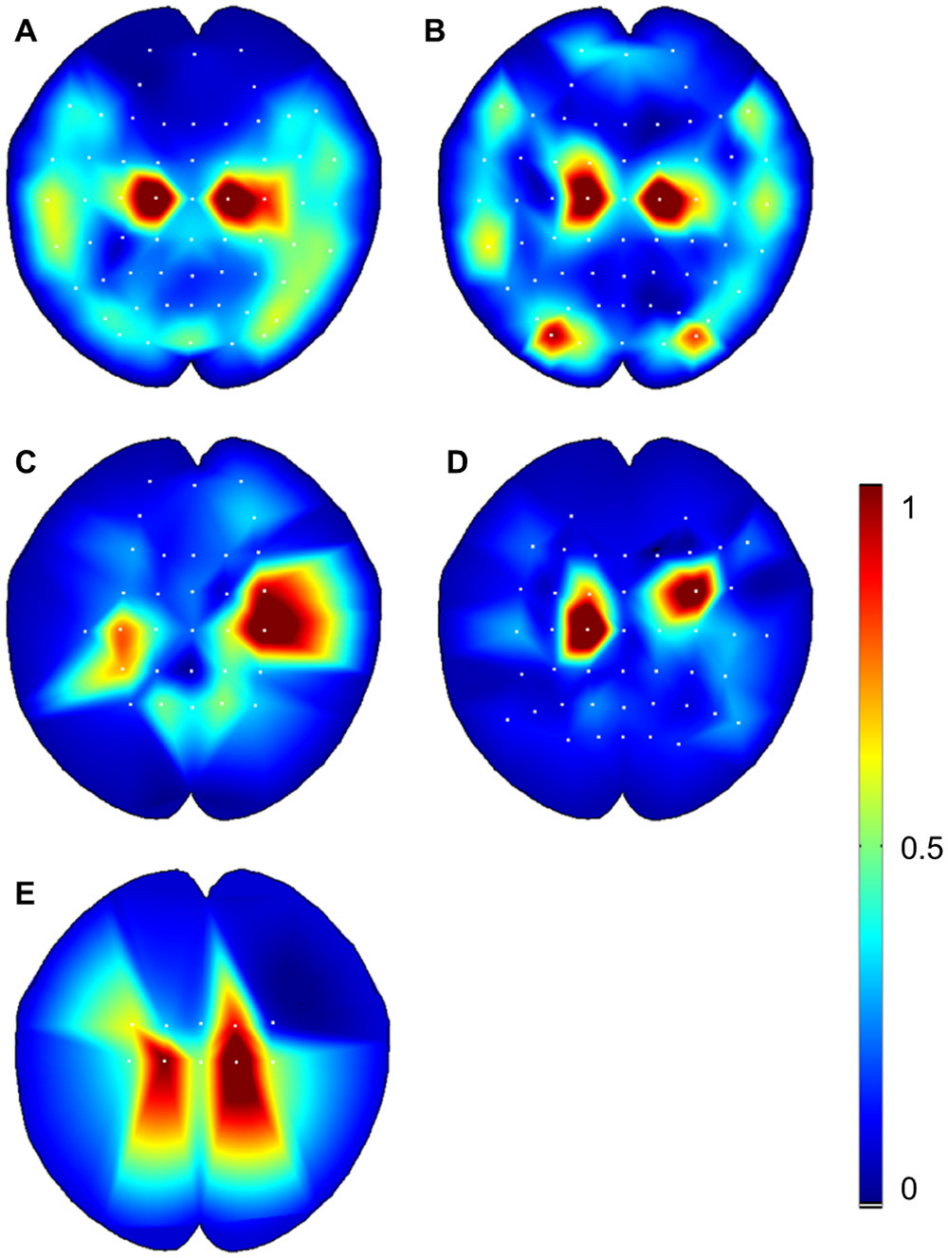
Spatial filtering of artificial neural networks (ANNs). The absolute weight values between the input and first hidden layer are averaged across time and over neurons in the first hidden layer to obtain a single value for each EEG electrode channel. The weights are then normalized so the heat map shows the minimum weight at 0 and the maximum weight at 1. The figure for each participant was obtained from the average weights over the five-folds of cross-validation **A** Subject A used 62 electrode channels. **B** Subject B used 62 electrode channels. **C** Subject C used 27 electrode channels. **D** Subject D used 46 electrode channels. **E** Subject E used 10 electrode channels.

Through analysis of network input layer weights, the influence of various spatial and temporal patterns can be inferred. Fig 9 shows the network input weights over all the cross validation partitions. The heat maps in Fig 9 are a mapping from the input layer to the first hidden layer of the network (see Fig 1) and thus represent a small part of the true interactions between the input signal and the output. The weights for each electrode channel were obtained by averaging the absolute input weights over time and across the number of neurons in the next layer. In this way, an implicit measure of the relative importance of each channel is obtained. For Fig 9A, 9B and 9D, the largest average weights were from the central and frontal-central group electrodes, which were placed above the motor cortex [23, 24]. In other words, the neural network classifiers extracted features with the greatest weighting from the signal sources above the motor cortex and pre-motor cortex [23, 24]. The same pattern is less evident in Fig 9C and 9E, as these participants used fewer electrode channels so the difference in spatial weighting is less pronounced.

**Fig 10:**
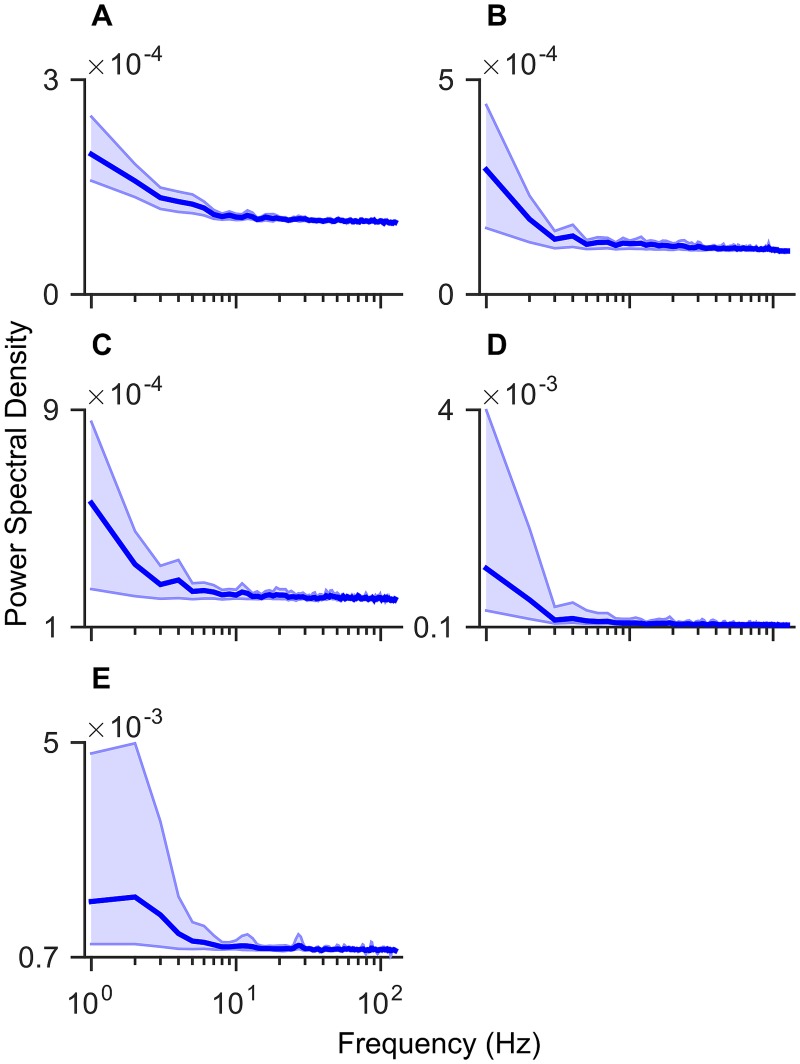
Temporal filtering of artificial neural networks (ANNs). Subplots are the estimated power spectral densities (PSDs) from the periodogram of the temporal weights. Temporal weights are taken between the input layer and the first hidden layer averaged over space (channels) and across the neurons in the first hidden layer to obtain a single value for each point in time. The initial weight values were obtained from the EEG epoch corresponding to each channel, with the DC component removed. The solid line represents the mean PSD over five folds of cross validation. The shaded region represents the distance between the minimum and maximum obtained values. **A** Subject A. **B** Subject B. **C** Subject C. **D** Subject D. **E** Subject E.

Fig 10 is an investigation of which frequencies in the EEG spectrum the ANNs emphasize. Emphasis is inferred using the average weight values between the input signal and each neuron in the first hidden layer, which are averaged over space and across the neurons in the first hidden layer. An interpretation of these weights is as finite impulse response filter coefficients acting on the input signal. Individual neurons may emphasize different spectral information and it is by using many of these individual filters that the ANN is able to construct complex features from high-dimensional data. It can be seen from Fig 10 that on average the lower frequencies appear to be accentuated by all the subjects ANNs. Although it is worthwhile to note that the analyses were averaged over many individual filters and do not represent temporal or spectral features created by the ANNs in and of themselves.

**Fig 11:**
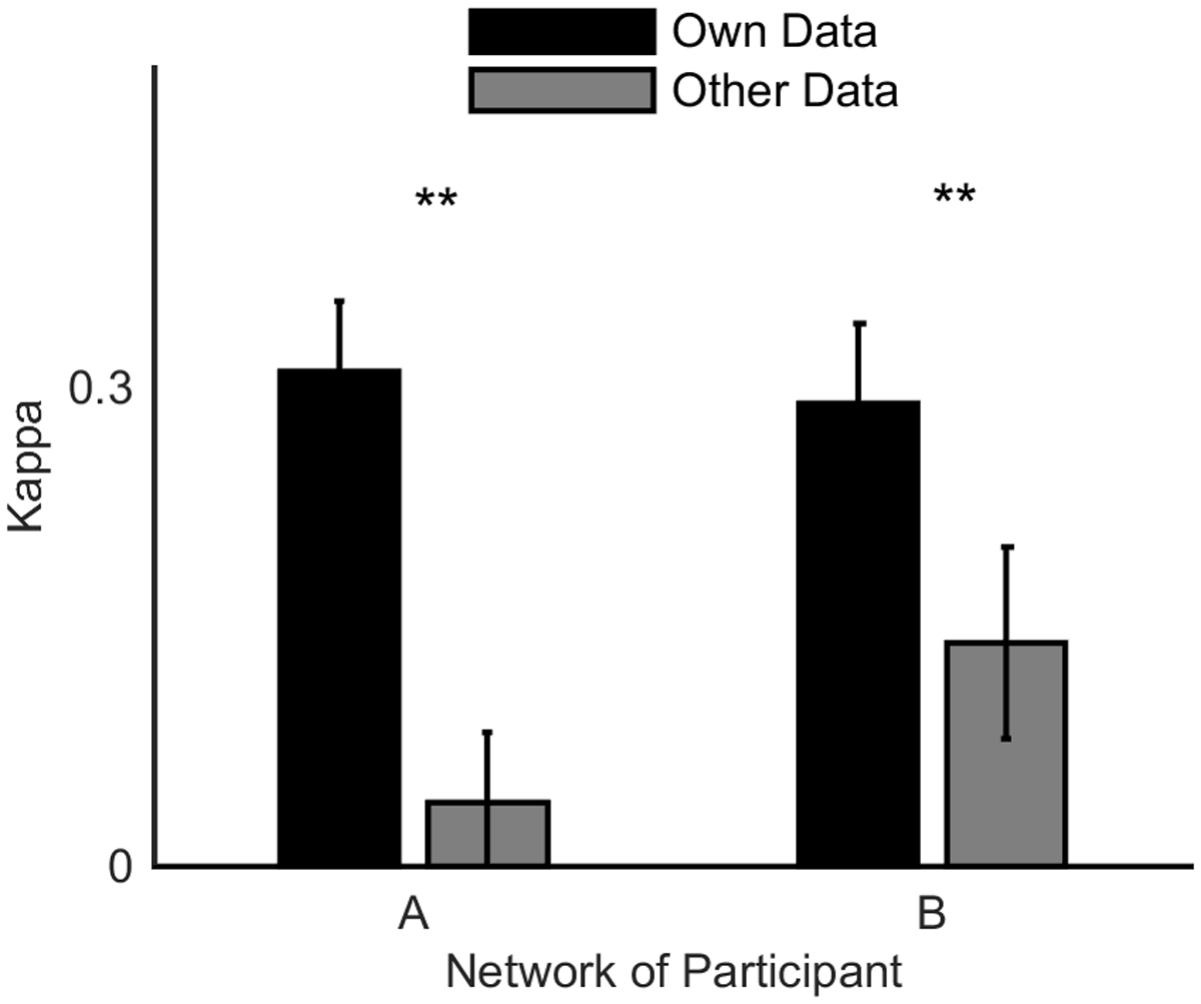
Inter-subject performance of two-class classifier. The classification performance (reported as the Cohen’s kappa score) of the artificial neural network (ANN) from Participant A is compared with test data from Participant B and vice versa. The bar plot shows the mean kappa value across the five-fold cross validation and the error bars are the standard deviation. The asterisks represent the confidence level to reject the null hypothesis that the results for the subject’s own dataset and the alternative dataset are not significantly different using a two-tailed *t*-test (** for *p* < 0.01).

In order to illustrate the subject-specific nature of the ANNs, the decoding algorithm for Participant A was tested with data from Participant B and vice-versa. The results of this test are shown in Fig 11. This analysis could not be completed for all participants, owing to differing numbers of recording electrodes. Comparison of Participant A’s results in Fig 11 shows that Participant A’s ANN performed significantly better for data from Participant A than Participant B (*p* = 1.05 × 10^−5^), using a two-tailed *t*-test (over the five-folds of cross validation). A statistically significant improvement was also obtained for Participant B (*p* = 0.0018). Fig 11 demonstrates that for Participants A and B, this method was capable of generating a subject-specific classifier that was tailored to the individual’s dataset and did not perform as well for a different participant.

## Discussion

We have demonstrated the feasibility of a new method for creating subject-specific motor brain-computer interfaces (BCIs) that do not rely on *a-priori* selection of features or recording channels. The novelty of the method is that the artificial neural network (ANN) structure can extract spatiotemporal features without network recurrence, so that backpropagation can still be used. A genetic algorithm (GA) was used to constrain a large number of candidate ANN solutions. A method of simulated annealing (SA) was used to augment and improve the network training.

The EEG signal is typically seen as an abstraction of the underlying neural activity and hence it is currently not possible to fully define all of the useful features present. The traditional approach to BCI design has been to rely on a few documented features that have demonstrated their usefulness empirically. However, there is a tendency amongst many of these features to generalize poorly between subjects and across different tasks. In light of this, a method that does not impose assumptions about the feature domain of the EEG signal is an improvement due to the potential for increasing the amount of information extracted from the signal. It is a potentially limiting approach to use a small number of features at the expense of ignoring all other possible information contained in the EEG signal. The results presented in this paper demonstrate that this new BCI method is able to accurately classify motor-related neurological signals without prior knowledge of task-specific features. Hence, this method has the potential to increase the performance of BCIs across a range of applications.

### Genetic Algorithm and Simulated Annealing Performance

The results in Fig 2 demonstrate that SA is beneficial, despite the added computational cost. There is greater standard deviation in fitness using backpropagation compared with the SA modified training algorithm. Hence, the SA modified training method produces more consistent results. Furthermore, the average Cohen’s kappa value increased using the SA alteration; that is, this method of training reaches a more optimal solution in the weight space of the network. The benefits of SA are less pronounced for the hand-squeeze datasets, which have many more training examples. This is to be expected as the more data that is available the better the expected performance using backpropagation for network training.

It was noted that the best performing ANNs selected from a randomly generated sample of 50 networks (seen in Fig 2) demonstrated similar levels of classification accuracy to the GA based selection. This is likely due to the large solution space which contains many ANNs capable of accurate classification (many local minima). The lack of convergence of the GA means that, in this case, the GA is performing similarly to a simple random search. Within the presented framework, there is scope to investigate alternative random search algorithms to constrain the ANNs, and the efficacy of such algorithms can be expected to improve in future.

The GA did not predominantly produce networks with maximal numbers of hidden layers or neurons, which is likely due to the potential for over-fitting with more complex networks. Fig 8 also shows that the GA identified ANNs with more hidden layers for the three-class dataset compared to two-class (Wilcoxon rank-sum test, *p* = 0.0094). This observation could indicate more layers are required for a more complex problem; however, the test only applies to this particular dataset and will not necessarily generalize. Drawing conclusions from the Wilcoxon rank-sum test would be more appropriate with an optimized solution, whereas the use of a GA here constrains rather than optimizes the ANN structure.

### Neural Network Analysis

The use of a neural network on such a high-dimensional problem has scope for improvement within the BCI research. A key issue is the unrealistically high volume of training data required to optimally train such a high-dimensional network. Despite the difficulty of converging to a single optimum weight-space of a network, the results demonstrate that an accurate classifier could be obtained for different subjects and tasks. Although the input dimension of the signal was orders of magnitude greater than the number of presented training samples, we have shown that the networks were capable of finding dominant spatial features to form a robust classifier. The advantage gained with a high-dimensional weight space is an increased capacity to learn unforeseen features that are unique to each data set and individual. Fig 9 showed that the networks placed higher weighting on the electrodes located over the motor and pre-motor cortex. This indicates that the ANNs were able to correctly predict the EEG channels most responsible for hand control during a motor task. This capability was demonstrated robustly over five-fold cross validation. The spatial weight patterns in Fig 9 were reasonably consistent between subjects; however, there was sufficient variation to suggest that for each participant a unique network was required, with the potential to extract distinct features. The temporal weights seen in Fig 10 demonstrated that the ANNs predominately emphasized the lower frequency content of the signal, which is a promising indication that the networks were learning to respond to task related EEG information, rather than high frequency artefact such as eye blinks or EMG from the brow, neck or jaw. The electrode heat-maps in Fig 9 also seem to indicate that the ANNs are not reliant these artefacts. A response to artefact could be indicated by high weights on the peripheral electrodes, which was seen to some extent for Participant B (Fig 9B).

There are a number of other examples in the literature involving the use of an ANN for classification of EEG signals; however, they typically rely on explicit feature extraction [11, 25–27]. The contribution of this work is in showing that the network does not require *a-priori* information about the neural signal for classification. The advantage of not performing extensive pre-processing of the data prior to inputting it to a network is that it enables the feature identification step to become automated. In addition, the novel architecture of the input layer enables temporal features to be extracted without requiring a more complex structure involving network recurrence or similar. This new method is valuable as there are a large number of potentially useful features, or combinations of features, and it is not possible to definitively determine the optimal one (or optimal set) for a given task. Therefore, a less constrained, black-box approach is well suited to the problem of feature selection for the EEG signal.

Future work could be undertaken in testing the efficacy of different machine learning classification techniques on time domain EEG data. Performance improvements could potentially be made using an adaptive network training algorithm that enables weight updates, or learning, to take place based on data acquired during real-time online use of a BCI. Other improvements may result from investigating alternative types of neural network. For example, deep-belief networks consisting of restricted Boltzmann machines (RBM) have shown promising results as a method of classifying of EEG signals [12]. The use of time-delay or recurrent neural networks could also be explored, as they are more robust to time shifts in the input signal classes [28]. Future work to improve the GA performance could investigate the use of other parameters in the genome, such as the start and end of the signal time window.

### Performance Comparison

The algorithm’s performance on open access BCI competition IV dataset three demonstrates that it is capable of performing well above chance outcome for a four class problem and shows comparable results with previously published studies, without relying on explicit feature extraction. This is a promising outcome, demonstrating that the classifier was capable of performing accurately despite the high dimensionality of the problem (only 160 training examples for a network with 4000 inputs). More recently, [20] reported new results using the same BCI IV competition data, reporting average test accuracy of 62% and 53% for two different classification methods. However, there is limited information available about how these results were obtained and hence it is difficult to make a detailed comparison. Krishna et al. [29] also reported improved classification accuracy on the same dataset, using a method of model fitting. The method presented here was also applied to BCI II competition data set four (Fig 4) and again demonstrated comparable results with published studies, despite using data obtained from a different experiment with a different signal acquisition method. More recently published studies showed improved accuracy of 84% [30] on the same BCI II competition dataset. These results represent a good performance benchmark; however, as they relied on significant signal processing in order to extract task-specific features, it is not clear how well the same algorithms would generalize to different tasks or signal types.

There are also a number of existing methods that aim to create generalizable classifiers for BCI applications. For instance Bai et al., [31] tested different combinations of spatio-temporal filtering and classification methods on a two-class, single-trial EEG motor-imagery task in order to tailor classifiers for 12 subjects. The highest accuracy (75%) was obtained using a combination of independent components analysis, power spectral density (PSD) estimation and support vector machine (SVM) [31]. A similar analysis also found the PSD and SVM to be most useful for a motor-imagery task, however in both cases it was noted that performance was highly subject-specific [31, 32]. Whilst different computational techniques may provide improved accuracy for different subjects or tasks, finding the optimal combination of feature and classifier may not be practical [31] so automating this process is worthwhile. The ability of ANNs to perform both implicit spatio-temporal feature extraction as well as classification on high dimensionality datasets is advantageous in this regard.

Although the current work does not demonstrate universally improved performance compared with all other available classifiers, it does provide a novel method of classification that is generalizable and does not rely on explicit *a-prioi* knowledge. Comparing performance based on a single dataset is not a rigorous validation of one method over another. However, the validation results on competition datasets demonstrate that this method is capable of performing similarly to other methods that were tailored for the specific problem, despite the different recording methods, task undertaken, number of classes and dimension of the datasets. Furthermore, the ability of this method to be applied across a range of tasks may enable the augmentation of existing classifiers, which are typically based on pre-defined features. There are undeniably many useful features that result in high levels of classification for different tasks. Combining the output of pre-existing methods with the approach outlined in this paper may enable increased performance compared to using either method alone. The information transfer rate of non-invasive BCIs has been largely unchanged for a decade [33]; therefore, a novel hybrid method combining physiological understanding and data-driven machine learning techniques warrants further investigation.

### Hand-Squeeze Classification Performance

For the dataset recorded for this study, both the two- and three-class methods resulted in classifiers that performed well above chance. The average Cohen’s kappa value across subjects for the two- and three-class classifiers were 0.58 and 0.37, respectively (where a kappa value of 0 corresponds to random guessing). The average accuracy on unseen test data was 78.9% and 60.7% for the two- and three-class classifiers, respectively. The classification accuracy was consistent across five subjects and was obtained from reasonably sized and well balanced datasets. A promising indication that the classifiers are subject specific is the fact that the performance for Participants A and B was significantly better for subject specific data (see Fig 11), despite the networks placing weighting in similar spatial locations (as seen in Fig 9). From these results, it would be anticipated that an online BCI is attainable given an appropriate interface between the classifier output and system response. However, it is important to note that there are additional challenges associated with translating offline classification of neural signals into online BCI use.

One difficulty in applying a classifier to online BCI use is that the offline performance classifying motor tasks of healthy subjects may not translate well to the neural signals of motor impaired users. A method of evaluating the anticipated online performance is to classify imagined tasks (motor imagery) rather than actual movements; however, typically participants require some training before they are able to reliably produce motor imagery related signals [34]. Furthermore, there is also evidence that the neural signals produced by a paralyzed user during attempted movements are as distinct as those of a healthy user performing the same motor task [35, 36]. In fact, instructing participants to imagine a motion but then prevent themselves actuating it, may actually have a confounding effect on the signal [21]. The potential differences between healthy and motor-impaired subjects provides further motivation to create a generalized method of classification.

As demonstrated in Figs 5 and 6, the classification accuracy for the two-class method was higher than the three-class method. A drop in accuracy as the number of classes increases is expected, as the probability of a classifier randomly selecting the correct class decreases. It must be noted that there are difficulties in drawing absolute comparisons based on accuracy alone between classifiers with differing numbers of classes. However, the Cohen’s kappa value takes this difficulty into account and it can be seen that the two-class method outperforms the three-class method (0.58 compared with 0.37). This is likely to be due to the fact that a class corresponding to no hand movements could contain many patterns of neural activity. The drop in classification performance may have been exacerbated by the likelihood that the user was still thinking about or planning hand movements even when they were not physically squeezing their hand, due to the repetitive nature of the training task. However, it is anticipated that using the classifier in a BCI, a three-class network will outperform the two-class network at recognizing the no-movement state, as the network has learnt defining features for this state whereas the two-class network is reliant on an absence of features pertaining to left or right hand squeezes.

It is clear from Fig 7 that once the three-class ANN has correctly identified a hand-squeeze the accuracy of left or right classification is improved. This indicates that the reduced kappa score of the three-class classifier compared with the two-class method is largely due to the difficulty of detecting the difference between a hand squeeze or no-movement class. However, it would be anticipated that the ability to distinguish between a hand squeeze and no-movement would be important to reduce the false positive rate in online use of the system. Therefore, in order to improve the overall performance of a three-class classifier, the main area to focus on is improving the quality and quantity of the examples for the no-movement class, so that these examples are more representative of a user’s brain signals during the idle-state. Experimental design modifications could be identified in order to ensure that the training data more closely represents the real-time use of the BCI. To an extent, the more data presented to a network for training, the better the performance will be. Hence, recording participant training data for a greater duration could potentially increase performance for both left or right hand squeeze or no-squeeze classifications. Due to the nature of neural networks, this system can be generalized to any number of output neurons and hence output commands. An avenue of investigation could be to use the numerical output of the final network layer to inform the velocity of an actuated device.

## Conclusion

The use of a high-dimension neural network has been investigated as a method for the classification of real-time EEG for use in a BCI. This method was validated on two separate datasets from previously held BCI competitions as well as EEG recordings from five participants. The explicit use of feature extraction before signal classification was not used in this application, instead a neural network was able to effectively act as both feature extractor and classifier. The method performed consistently well across different signal types and experimental tasks. The results demonstrate an ability to extract task-related features despite the high dimensional input data. The results presented are an indication that a high-dimensional machine learning method is a viable alternative to explicit feature extraction, despite the relatively high computational costs involved during training. The benefits of this method are the increased generalizability to different types of control task, signal acquisition methods and between subjects. The independence of the system from pre-defined features provides greater potential to tailor a BCI to an individual, and may also reveal previously undiscovered features of neural signals.

## Supporting Information

S1 Text Cohen’s Kappa Calculation. (PDF)

S1 Appendix Confusion Matrices and Artificial Neural Network Sizes. (PDF)

S2 Appendix Exact p-values for Figs 2, 5 and 6. (PDF)
