# Supplementary material for: A Generalizable Brain-Computer Interface (BCI) Using Machine Learning for Feature Discovery
Source: PLoS One. 2015 Jun 26;10(6):e0131328. doi: 10.1371/journal.pone.0131328 (PMC4482677; doi:10.1371/journal.pone.0131328)
Supplement: S1 Text — (PDF) [file pone.0131328.s001.pdf]

S1 Text - Cohen's Kappa Calculation

Cohen's kappa [1] value is given by

$$\kappa = \frac{p_a - p_e}{1 - p_e}, \tag{1}$$

where p_a is the accuracy and p_e is the probability of a chance detection for the given data set. For an M class problem with N trials

$$p_a = \frac{1}{N} \sum_{i=1}^{M} C_{i,i} \tag{2}$$

and

$$p_e = \frac{1}{N^2} \sum_{i=1}^{M} C_{i,:} C_{:,i}, \tag{3}$$

where C is the $N \times M$ confusion matrix and $C_{i,:}$ and $C_{:,i}$ represent the sum of elements in columns and rows of C (indexed by i), resepectively. p_e can be interpreted as normalizing the accuracy from the range $[0\ 1]$ to $[p_{\text{chance}}\ 1]$, where p_{chance} is the accuracy expected by random guess given a test subset (i.e. $\frac{1}{M}$ in an M-class classifier). Therefore, Cohen's kappa takes values of $[-1\ 1]$, where 0 represents the expected performance of a random guess, 1 represents perfect accuracy, and -1 represents perfect and consistent disagreement with the true values.

To test for significance, first the variance of κ is calculated as

$$var(\kappa) = \frac{p_e}{N(1 - p_e)} \tag{4}$$

From this, the standard z-score is calculated as

$$z = \frac{\kappa}{var(\kappa)} \tag{5}$$

from which a p-score is evaluated from the unit Gaussian distribution.

References

1. Cohen J. Weighted kappa: Nominal scale agreement provision for scaled disagreement or partial credit. Psychological bulletin. 1968;70(4):213–220.
