# Supplementary material for: A Generalizable Brain-Computer Interface (BCI) Using Machine Learning for Feature Discovery
Source: PLoS One. 2015 Jun 26;10(6):e0131328. doi: 10.1371/journal.pone.0131328 (PMC4482677; doi:10.1371/journal.pone.0131328)
Supplement: S1 Appendix — (PDF) [file pone.0131328.s002.pdf]

S1 Appendix - Confusion Matrices and Artificial Neural Network Sizes

Average of Five-Fold Cross Validation Confusion Matrices

The results of the three-class classifier for each participant are presented as confusion matrices. Each is the average of the five-fold cross validation. The rows of the table represent the actual class and the columns show the predicted class as output by the ANN classifier. Results are presented as percentages and standard deviations. Values within the confusion matrix are the average percentage of entries within that cell. Percentages at the end of the rows and columns indicate the percentage of entries in that row/column that are correct classifications. The bottom right value is the overall accuracy (the sum of the diagonal entries).

Table 1. Average results of five-fold cross validations for Participant A

		Predicted			
		No Squeeze	Right	Left	Sensitivity
Actual	No Squeeze	35.5% ± 1.0	10.4% ± 1.8	13.1% ± 1.2	60.2% ± 1.4
	Right	8.1% ± 2.1	10.7% ± 1.9	2.4% ± 1.1	50.6% ± 6.2
	Left	5.9% ± 1.4	2.0% ± 0.7	12.0% ± 2.0	60.3% ± 8.2
	Specificity	72.0% ± 4.5	46.4% ± 6.9	43.6% ± 6.1	**58.2% ± 2.2**

Table 2. Average results of five-fold cross validations for Participant B

		Predicted			
		No Squeeze	Right	Left	Sensitivity
Actual	No Squeeze	35.2% ± 1.8	8.4% ± 1.8	9.6% ± 1.5	66.2% ± 1.1
	Right	9.7% ± 3.4	11.0% ± 1.0	2.8% ± 1.9	48.4% ± 11.3
	Left	9.8% ± 2.5	2.6% ± 1.8	10.9% ± 0.7	47.0% ± 4.5
	Specificity	64.4% ± 5.0	51.0% ± 7.3	47.0% ± 5.8	**57.1% ± 2.9**

Table 3. Average results of five-fold cross validations for Participant C

		Predicted			
		No Squeeze	Right	Left	Sensitivity
Actual	No Squeeze	24.8% ± 4.2	6.5% ± 1.6	5.4% ± 1.8	67.4% ± 4.7
	Right	13.2% ± 2.4	17.5% ± 3.9	1.3% ± 0.4	54.4% ± 8.9
	Left	14.8% ± 2.1	3.3% ± 1.0	13.4% ± 4.0	42.0% ± 8.2
	Specificity	47.0% ± 3.7	63.6% ± 6.9	66.4% ± 6.8	**55.6% ± 3.2**

Table 4. Average results of five-fold cross validations for Participant D

		Predicted			
		No Squeeze	Right	Left	Sensitivity
Actual	No Squeeze	23.7% ± 2.7	8.4% ± 2.2	5.0% ± 1.4	63.7% ± 4.7
	Right	9.8% ± 2.7	19.1% ± 2.0	1.9% ± 0.6	62.0% ± 6.3
	Left	9.0% ± 1.6	1.9% ± 1.4	21.1% ± 3.3	65.8% ± 8.2
	Specificity	55.8% ± 3.7	65.3% ± 8.5	75.1% ± 4.1	**63.9% ± 4.3**

Table 5. Average results of five-fold cross validations for Participant E

		Predicted			
		No Squeeze	Right	Left	Sensitivity
Actual	No Squeeze	38.9% ± 3.2	5.5% ± 1.4	7.4% ± 1.4	75.1% ± 3.1
	Right	6.6% ± 1.0	15.6% ± 2.0	2.8% ± 0.8	62.5% ± 6.1
	Left	6.1% ± 1.2	3.0% ± 0.9	14.1% ± 1.3	60.7% ± 4.0
	Specificity	75.3% ± 2.5	64.9% ± 5.6	57.9% ± 3.8	**68.6% ± 0.8**

ANN Hidden Layers - 2 class

Table 6. Hidden Layer Configuration for Participant A's ANN

Validation Fold	Hidden Layers
One	80-213-20
Two	281
Three	191
Four	6
Five	101-493-207

Table 7. Hidden Layer Configuration for Participant B's ANN

Validation Fold	Hidden Layers
One	96
Two	154
Three	101
Four	493
Five	104

Table 8. Hidden Layer Configuration for Participant C's ANN

Validation Fold	Hidden Layers
One	287
Two	366
Three	122
Four	295
Five	375

Table 9. Hidden Layer Configuration for Participant D's ANN

Validation Fold	Hidden Layers
One	4-361
Two	441-70
Three	154-333-173
Four	78-238-13
Five	10

Table 10. Hidden Layer Configuration for Participant E's ANN

Validation Fold	Hidden Layers
One	59
Two	31
Three	280
Four	237
Five	361

ANN Hidden Layers - 3 class

Table 11. Hidden Layer Configuration for Participant A's ANN

Validation Fold	Hidden Layers
One	34
Two	69-144
Three	114-287
Four	464-17
Five	453-81

Table 12. Hidden Layer Configuration for Participant B's ANN

Validation Fold	Hidden Layers
One	343-54
Two	295-40
Three	474
Four	255
Five	328

Table 13. Hidden Layer Configuration for Participant C's ANN

Validation Fold	Hidden Layers
One	477-290-15
Two	71-157-18
Three	197-3
Four	423-54-257
Five	324-29

Table 14. Hidden Layer Configuration for Participant D's ANN

Validation Fold	Hidden Layers
One	115-261-21
Two	440
Three	105-171
Four	79-290
Five	250-28-192

Table 15. Hidden Layer Configuration for Participant E's ANN

Validation Fold	Hidden Layers
One	147
Two	94-479
Three	411
Four	308
Five	205-307-205
