# Supplementary material for: A Generalizable Brain-Computer Interface (BCI) Using Machine Learning for Feature Discovery
Source: PLoS One. 2015 Jun 26;10(6):e0131328. doi: 10.1371/journal.pone.0131328 (PMC4482677; doi:10.1371/journal.pone.0131328)
Supplement: S2 Appendix — (PDF) [file pone.0131328.s003.pdf]

S2 Appendix - Exact p-values for Figures 2, 5 and 6

Table 1. Exact p-values for Figure 2

Dataset	p-value
two-class hand squeeze	1.04×10^{-22}
three-class hand squeeze	6.90×10^{-22}
BCI Competition II	1.83×10^{-13}
BCI Competition IV	7.06×10^{-16}

The table shows the exact calculated p-vaues for the comparison of backpropagation (BP) and simulated annealing augmented BP to train an artificial neural network (ANN). Each method was used to train a randomly initialized one-, two- or three-layer ANN and this was repeated 50 times. A two-tail t-test was used to determine whether the two sets of Cohen's kappa values were significantly unlikely to be produced by chance if the means of the two distributions were equal (over the sample size of 50). Note that p-values were calculated using MATLAB function *ttest2* with 32-bit precision.

Table 2. Exact p-values for Figure 5

Participant	p-values for five folds of cross-validation				
A	0.72×10^{-5}	0.2×10^{-8}	0.5×10^{-8}	0.01×10^{-5}	0.31×10^{-7}
B	0.18×10^{-7}	0.53×10^{-8}	0.4×10^{-6}	0.19×10^{-8}	0.31×10^{-11}
C	0.1×10^{-10}	0.3×10^{-14}	0.69×10^{-13}	0	0.22×10^{-11}
D	0	0	0	0	0
E	0.15×10^{-11}	0	0	0	0.36×10^{-13}

The table shows the exact calculated p-vaues for the comparison of the Cohen's kappa values and chance performance for the two-class hand squeeze dataset. For further details on Cohen's kappa and the significance calculation see S1 Text. Note that p-values were calculated using MATLAB with 32-bit precision.

Table 3. Exact p-values for Figure 6

Participant	p-values for five folds of cross-validation				
A	0.23×10^{-8}	0.22×10^{-10}	0.19×10^{-6}	0.1×10^{-9}	0.3×10^{-14}
B	0.64×10^{-7}	0.82×10^{-7}	0.23×10^{-11}	0.13×10^{-9}	0.28×10^{-7}
C	0.35×10^{-8}	0	0.62×10^{-8}	0.47×10^{-13}	0.13×10^{-12}
D	0	0	0	0	0
E	0	0	0	0	0

The table shows the exact calculated p-vaues for the comparison of the Cohen's kappa values and chance performance for the three-class hand squeeze dataset. For further details on Cohen's kappa and the significance calculation see S1 Text. Note that p-values were calculated using MATLAB with 32-bit precision.
